# Supplementary material for: Patterns of Intron Gain and Loss in Fungi
Source: PLoS Biol. 2004 Nov 30;2(12):e422. doi: 10.1371/journal.pbio.0020422 (PMC532390; doi:10.1371/journal.pbio.0020422)
Supplement: Table S1 — Also available at http://genes.mit.edu/NielsenEtAl/. (4.3 MB ZIP). [file pbio.0020422.st001.zip › NielsenEtAl/html/743.html]

AN1689.1.NCU00378.1.MG05008.1.FG02273.1


```
 CLUSTAL W (1.82) Multiple Sequence Alignments - Introns Inserted


Sequence 1: NCU00378.1	495 aa
Sequence 2: MG05008.1	497 aa
Sequence 3: AN1689.1	501 aa
Sequence 4: FG02273.1	500 aa
Alignment Length: 501 aa
Number Identitical Residues: 269 aa
Alignment Score (without introns) 13186


MG05008.1 	--MNIQLSAPNGKKWIQPTGLFINNEFVKSSNGQKLTTVNAA~TEEDITSVYSATEEDID
NCU00378.1	--MEVELTAPNGKKWMQPLGLFINNEFVKSANEQKLISINPT2TEEEICSVYAATAEDVD
FG02273.1 	MSLDIQLTAPNGRTYKQPTGLFINNEWVKSSNGEKITSINPT2NEQEITSVYAGSSEDVD
AN1689.1  	MATAVSLTAPNGHKYEQPIGLFINNEFVASKSGEKFATVNPS2DEEEITQVYAAGEEDID
          	 :  :.*:****:.: ** *******:* * . :*: ::*.:  *::* .**:.  **:*

MG05008.1 	KAVKAARAAFKHPSWRNLSGTERGEMMHKLADLVVKNAETLATIEALDGG~KPYSVALGE
NCU00378.1	AAVSAARKAFRHESWKSLSGTERGALMRKLADLVAENAEILATIECLDNG~KPYQTALNE
FG02273.1 	KAVRAARRALHNPSWRDLPGTERGKLLSRLATLVEENKEILATIETWDNG~KPYTVALND
AN1689.1  	IAVKAARKALKDPSWKLLTATDRGNLMLKLADLIDQNKETLAVIETWDNG1KPYQVSLND
          	 ** *** *::. **: *..*:** :: :** *: :* * **.**  *.* *** .:*.:

MG05008.1 	DVVEFEKTIRYYAGFADKNFGQTIDVGPNKFAYTIKEPIGVCGQIIP~WNYPLCMAAWKL
NCU00378.1	NVPEVINVLRYYAGYADKNFGQVIDVGPAKFAYTVKEPLGVCGQIIP~WNYPLDMAAWKL
FG02273.1 	DVNEVAETLRYYGGFADKVYGQVIETTGDKFGYTIREPIGVCGQIIP2WNFPLAMAAWKL
AN1689.1  	DLSEVVNTIRYCAGWADKIHGQTISTTPAKFAYTLRQPIGVVGQIIP~WNFPLAMAAWKL
          	:: *. :.:** .*:*** .**.*..   **.**:::*:** ***** **:** ******

MG05008.1 	GPALACGNTVVLKLAEQTPLSMLYVATLIKEAGFPAGVVNVINGLGGEAGAAIVQHPDID
NCU00378.1	GPALCCGNTVVLKLAEQTPLSVLYLAKLIKEAGFPPGVINIINGHGREAGAALVQHPQVD
FG02273.1 	GPALACGNAVILKPAEQTPLSILYLANLIVEAGFPPGVVNIINGLGTVAGAALASHMDVD
AN1689.1  	GPALACGNTVVLKPAEQTPLSILYLAKFIKEAGFPPGVVNIVNGLGRVAGSALVTHPGVD
          	****.***:*:** *******:**:*.:* *****.**:*::** *  **:*:. *  :*

MG05008.1 	KVAFTGSTATGKQIMKACAGTMKNITLETGGKSPLLVFDDADLDLAAEWSHIGIMSNQGQ
NCU00378.1	KIAFTGSTTTGKEIMKMASYTMKNITLETGGKSPLIVFEDADLELAATWSHIGIMSNQGQ
FG02273.1 	KIAFTGSTPTARSIMKMASSNLKNITLETGGKSPLIVFDDADVDLAAYWAHAGIMYNQGQ
AN1689.1  	KVAFTGSTMTGKEIMKMAAGTMKNVTLETGGKSPLLVFDDADLEQAAKWAHIGIMYNQGQ
          	*:****** *.:.*** .: .:**:**********:**:***:: ** *:* *** ****

MG05008.1 	ICTATSRILVHESVYDKFVGRFKDTVEKVSIVGDPFDEKTFQGPQVTKAQYEKVLGYIKL
NCU00378.1	ICTATSRILVHEKIYDEFVEKFKAKVQEVSVLGDPFEESTFHGPQVTKAQYERVLGYINV
FG02273.1 	VCTATSRILVQESIYDKFMAAFGAQVKNISKVGDPFEESTFQGPQVTKAQYERILSFVDV
AN1689.1  	VCTATSRILVHEKVHDEFIRLFREAVATTSKVGDPFSDDTFQGPQVTKAQYERVLSYIES
          	:*********:*.::*:*:  *   *   * :****.:.**:**********::*.::. 

MG05008.1 	AQEEGATIATGGKPAP--QNGKGFYVAPTVFTNVKTSMRVYKEEIFGPCVSIISFKDEEE
NCU00378.1	GKEEGATVMMGGEPAP--QNGKGFFVAPTVFTNVKPTMKIFREEIFGPCVAITTFKTEEE
FG02273.1 	GKKEGAKLALGGQPFK-VGDGKGYFVEPTVFTDVTPKMRVFQEEVFGPFVVVTKFSSEDE
AN1689.1  	GKQEGATLVDGGVPYKNVKDGKGFFIAPTIFTNVKDNMRIYREEVFGPFVAIARFSTEEE
          	.::***.:  ** *  .  :***::: **:**:*. .*::::**:*** * :  *. *:*

MG05008.1 	AVELANDTTYGLGAAVFTRNLTRAHRVAREIQSGMVWINSSNDSDCRVPFGGVKQSGIGR
NCU00378.1	ALTLANDSMYGLGAALFTKDLTRAHRVAREIEAGMVWVNSSNDSDFRIPFGGVKQSGIGR
FG02273.1 	AIHLANDTQYGLGSALFTTNLTRAHQVAKRIEAGMVWVNSSNDSDWRIPFGGVKQSGIGR
AN1689.1  	AIDRANDTTYGLGAAVFTKDIERAHRVASEIEAGMVWINSSNDSDFRVPFGGVKQSGIGR
          	*:  ***: ****:*:** :: ***:** .*::****:******* *:************

MG05008.1 	ELGEAGLASYCNIKSVHVNMAAHL
NCU00378.1	ELGEAGLAPYCNVKSIHVNLAA--
FG02273.1 	ELGEAGLAAYSNIKAVHVNIAAKL
AN1689.1  	ELGEAGLEAYTQIKAVHVNMGTKL
          	******* .* ::*::***:.:
```
